# Supplementary material for: Evaluation and characterization of expression quantitative trait analysis methods in the Hybrid Rat Diversity Panel
Source: Front Genet. 2022 Sep 14;13:947423. doi: 10.3389/fgene.2022.947423 (PMC9515987; doi:10.3389/fgene.2022.947423)
Supplement: Supplementary file 1 [file DataSheet1.PDF]

Supplementary Material for 'Evaluation and characterization of expression quantitative trait analysis methods in the Hybrid Rat Diversity Panel'

Jack Pattee, Lauren A. Vanderlinden, Spencer Mahaffey, Paula Hoffman, Boris Tabakoff, Laura M Saba

April 2022; revised August 2022

S1 Simulation Study Design

This supplementary section describes the design of the simulation study and some preliminary results involving the simulated data. We extensively detail the simulation method and present some results indicating that the simulated data coheres well to the real data.

A general overview of the simulation setup is as follows. We used real genetic marker data from 92 HRDP subjects genotyped on 18,342 SNPs in the simulation. After simulating SNP effects in the manner described throughout this supplementary section, we simulated phenotypes by matrix multiplication between the real genotype data and the simulated SNP effects. While data are simulated using the entire set of 18,342 SNPs, only SNPs that pass the quality control described in the manuscript (i.e., minor allele frequency $> .1$, missinginess $< 5\%$) are included as fixed effects in the inferential model. Broadly, there are two analyses we are interested in: the set of 43 subjects for which we have real data ('43-subject simulations') and the full set of 92 subjects and the three subpopulations ('subpopulation simulations'). In the 43-subject simulation, we apply the GEMMA LOCO, standard GEMMA, and subpopulation meta-analysis methods. In the subpopulation simulations, we apply only GEMMA LOCO. The intention of the 43-subject simulations is to establish the power and type I error of the three competing methods. The intention of the subpopulation simulations is to compare the power of GEMMA LOCO to detect SNP effects in the full HRDP panel to each of the subpopulations.

The simulation study involved three unique simulation settings, as follows:

1. 'No cis effect' or 'unimodal' simulation, where there is no difference in effect size between cis SNPs and trans SNPs.
2. 'One cis effect' or 'bimodal one cis' where cis SNPs contribute to a substantially larger proportion of the heritability than trans SNPs, and exactly 1 cis-SNP has nonzero effect size.
3. 'Multiple cis effects' or 'bimodal multiple cis', where cis-SNPs contribute to a substantailly larger proportion of the heritability than trans SNPs, and the number of cis-SNPs with nonzero effect size is simulated from a binomial distribution, with mean number of nonzero cis-SNPs equal to two.

These three simulation protocols are used to generate simulated phenotypes for the full set of 92 HRDP subjects. The subsequent analyses, i.e. the 43-subject simulations and the subpopulation simulations, are conducted by appropriately subsampling from the full genotype and simulated phenotype data. For the 43 subject simulations, we apply the standard GEMMA, GEMMA LOCO, and subpopulation meta-analysis methods to each of the three simulation protocols, which is a total of 9 analyses. For the subpopulation simulations, we apply the GEMMA LOCO model to each simulation setting in each of the following four populations: all 92 subjects, only recominant HXB/BXH, only recominant FXLE/LEXF, only classic inbred. This is a total of 12 analyses. Table S1 describes the full set of 21 analyses conducted.

	No cis effect	One cis effect	Multiple Cis Effect
43-Subject	GS, GL, SM	GS, GL, SM	GS, GL, SM
Full HRDP (92 Strains)	GL	GL	GL
HXB/BXH (30 Strains)	GL	GL	GL
FXLE/LEXF (33 Strains)	GL	GL	GL
Classic Inbred Only (29 Strains)	GL	GL	GL

Table S1: Analyses performed for each population and each simulation setting. **GS** signifies 'GEMMA Standard', **GL** signifies 'GEMMA LOCO', **SM** signifies 'Supopluation meta-analysis'

Each simulated gene expression was simulated to have heritability equal to the (GEMMA estimated) heritability of the gene from the real data. The correspondence is, of course, not exact due to stochasticity. For the no cis effect simulation, we used only genes estimated to be unimodal in the real data. For the two bimodal simulations, we used only genes estimated to be bi- or tri-modal in the real data. In the following three subsections, we present some results to confirm that our setup is working as intended. The following three subsections describe the three simulation settings in detail.

S1.1 No cis effect simulation

For the unimodal simulation, we simulate SNP effects from a point-normal distribution as follows, for all $j \in [1, \ldots, 18, 342]$ SNPs:

$$\beta_j \sim_{iid} \begin{cases} N(0, \frac{h^2}{p \times M}), \text{ with probability p} \\ 0, \text{ with probability 1-p} \end{cases}$$

We fix $p = 1/10$, which entails that 10% of SNPs have a heritable nonzero contribution to the phenotype. M is set equal to the number of SNPs (18,342). h^2 is the chip-based hertiability from the gene estimated via standard GEMMA on the real data. We simulated phenotypes for a random sample of 1000 genes drawn from the 81,992 genes that were estimated to be unimodal in the real data. After simulating SNP effects from the above point-normal model, we have the heritable portion of the phenotype, which we can denote as $\mathbf{X}\boldsymbol{\beta}$. Note here $\mathbf{X}$ is the genotype matrix of SNP data. We then simulate error variance according to the following distribution: $\boldsymbol{\epsilon} \sim N(0, \frac{(1-h^2) \times var(\mathbf{X}\boldsymbol{\beta})}{h^2}\mathbf{I})$ and define our gene expression phenotype $\mathbf{y} = \mathbf{X}\boldsymbol{\beta} + \boldsymbol{\epsilon}$. We uniquely simulate phenotypes for 1000 genes in this manner. Note that it is possible for a cis-SNP to be causal in this simulation setup, somewhat contrary to the 'no cis effect' nomenclature. It is the case that cis-SNPs are not simulated to have a larger effect than trans SNPs. Any given SNP is causal with probability $p = 1/10$, and as such cis-SNPs are occasionally causal in this framework.

We are interested in whether the simulated data coheres well to the real data, which is investigated as follows. Firstly, we see how well simulating in this manner generates a distribution of heritability that matches the true per-gene heritability. Figure S1 shows that the simulated heritability (calculated here as $cor(\mathbf{X}\boldsymbol{\beta}, \mathbf{y})^2$) is similar to the true heritability. Secondly, we investigate whether simulating gene expression in this fashion produces gene expression phenotypes that are truly unimodal. We used the R package mclust to estimate modality for the 43-subject simulation in the manner as for the real data, i.e. fitting uni, bi, and trimodal models and selecting the optimal model via BIC. Figure S2 displays density plots and histograms for three randomly chosen genes, and table S2 displays a count of the number of genes with each modal assignment. These results indicate that this simulation setting does a reasonable job of inducing unimodality in the simulated phenotype.

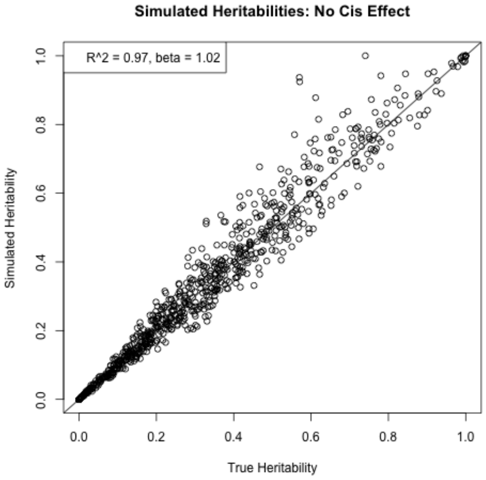

Figure S1: Simulated chip-based heritability versus true chip-based heritability for 1000 simulated unimodal genes.

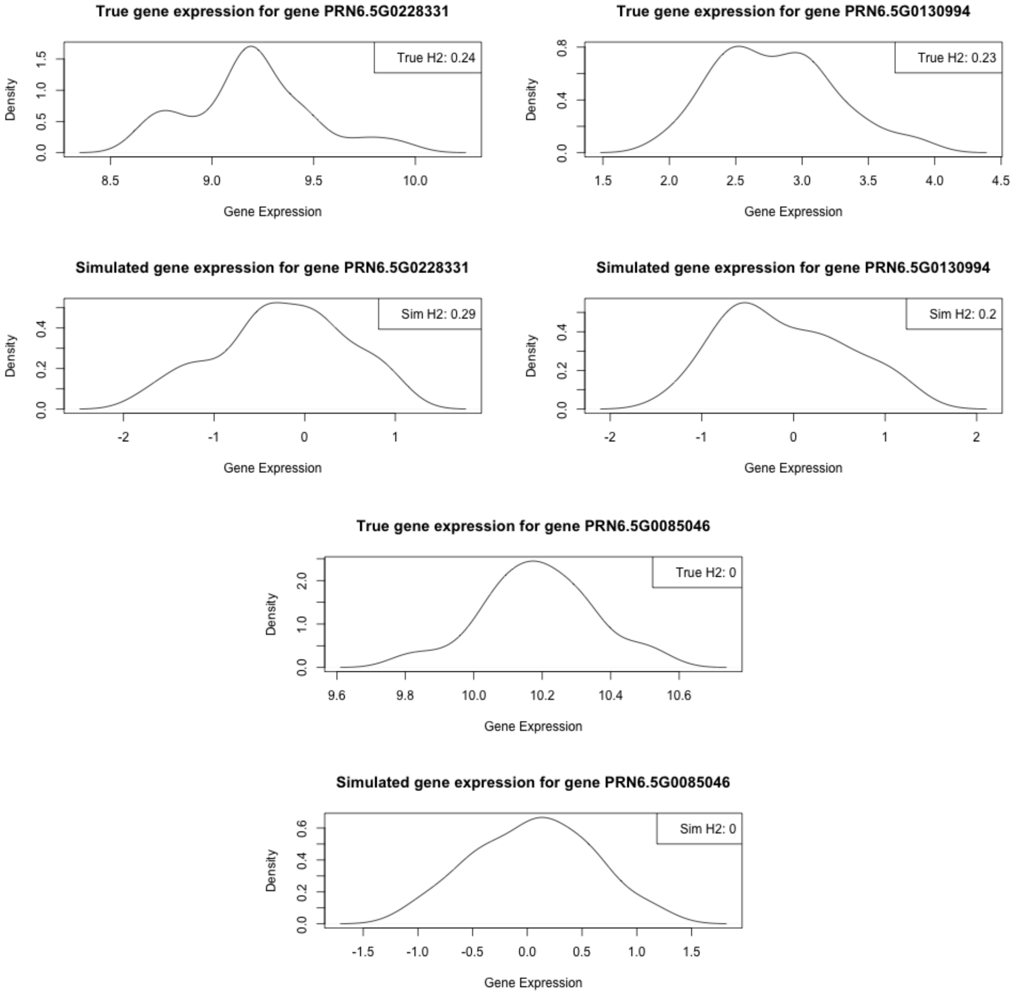

Figure S2: Distribution of simulated versus true gene expression for three randomly chosen unimodal genes (out of 1000).

Cluster Assignments	Unimodal	Bimodal	Trimodal
Count of Genes	954	42	4

Table S2: Count of genes per number of modes as estimated by 'mclust', for unimodal simulation.

S1.2 One cis effect simulation

For the one cis effect simulation, we simulate phenotypes for a randomly chosen set of 1,000 genes out of the 17,384 genes that are estimated to be bimodal or trimodal in the real data. To induce bimodality in our simulated phenotypes, we simulate SNP effects for the cis and trans regions from different point-normal distributions. We fix the proportion of the total heritability attributable the cis region at T (denoting 'heritability Tradeoff'). For each of the 1000 genes that we simulate, we draw T from a $Unif(.7, .9)$ distribution. Consider that we have SNPs indexed by $i \in [1, \ldots, q_{cis}]$, where q_{cis} is the number of cis-SNPs. Let s be some randomly chosen index such that $1 \leq s \leq q_{cis}$. We then define the point-normal distribution for the cis-SNPs as follows:

$$\beta_j \sim_{iid} \begin{cases} N(0, \frac{h^2_{cis}}{p \times q_{cis}}), \text{for SNP} j = s \\ 0, \text{for all SNPs } j \text{ such that } j \neq s \end{cases}$$

In this way, we ensure that exactly one (randomly chosen) cis-SNP has nonzero effect size. We define $p = 1/q_{cis}$. h^2_{cis} is defined as the true heritability of the gene (estimated from real data) multiplied by T: that is, $h^2_{cis} = T \times h^2$.

Defining the distribution for the trans-SNPs is more difficult. Ultimately, we want that $h^2_{trans} + h^2_{cis} = h^2$. In order for this expression to hold, we need to ensure that the heritability contributed from the cis-SNPs and the trans-SNPs is orthogonal. To ensure this, we only simulate nonzero effects for trans-SNPs that are on different chromosomes than the gene in question. That is, all trans SNPs on the same chromosome as the gene have effect set equal to zero. We also want that $\frac{h^2_{cis}}{h^2} = T$. If we partition $\boldsymbol{\beta}$ into cis and trans components $\boldsymbol{\beta}_{trans}$ and $\boldsymbol{\beta}_{cis}$ and do likewise with the genotype matrix $\mathbf{X}_{trans}$ and $\mathbf{X}_{cis}$, we can define the cis heritability as follows:

$$h^2_{cis} = \frac{var(\mathbf{X}_{cis}\boldsymbol{\beta}_{cis})}{var(\mathbf{X}_{cis}\boldsymbol{\beta}_{cis}) + var(\mathbf{X}_{trans}\boldsymbol{\beta}_{trans}) + var(\boldsymbol{\epsilon})}$$

Here, we see the difficulty in defining a distribution for the trans effects: namely, that the variance of $\mathbf{X}_{trans}\boldsymbol{\beta}_{trans}$ depends on the distribution of the specific genotype matrix $\mathbf{X}_{trans}$ for that gene. We approach this as follows. We initialize the trans effects according to the point normal distribution as follows:

$$\beta_j \sim_{iid} \begin{cases} N(0, \frac{h^2_{trans}}{p \times q_{trans}}), \text{ with probability p} \\ 0, \text{ with probability 1-p} \end{cases}$$

With $p = 1/10$ and q_{trans}, defined as the count of all SNPs not on the same chromosome as the gene in question. Given this variance, we simulate SNP effects, and calculate $var(\mathbf{X}_{trans}\boldsymbol{\beta}_{trans})$. If the following expression is satisfied:

$$var(\mathbf{X}_{trans}\boldsymbol{\beta}_{trans}) < \frac{(1 - T) \times var(\mathbf{X}_{cis}\boldsymbol{\beta}_{cis})}{T}$$

We use the simulated $\boldsymbol{\beta}_{trans}$. Otherwise, we divide the variance of the effect size distribution $\frac{h^2_{trans}}{Mp}$ by 2, and simulate trans effects again until the condition is satisfied.

Once this is done, we can simulate normal errors such that the following expression holds:

$$h^2 = \frac{var(\mathbf{X}_{cis}\boldsymbol{\beta}_{cis}) + var(\mathbf{X}_{trans}\boldsymbol{\beta}_{trans})}{var(\mathbf{X}_{cis}\boldsymbol{\beta}_{cis}) + var(\mathbf{X}_{trans}\boldsymbol{\beta}_{trans}) + var(\boldsymbol{\epsilon})}$$

Figure S3 demonstrates that simulating effect sizes in the specified manner generates genes with roughly the desired heritability. Figure S4 displays the distribution of the simulated gene expression for three randomly selected bimodal genes versus the true distribution of the gene expression. Table S3 displays the estimated modality of the simulated phenotypes for the 43-subject simulation. We see that this simulation setting induces some amount of bimodality, but a majority of genes are estimated to be unimodal.

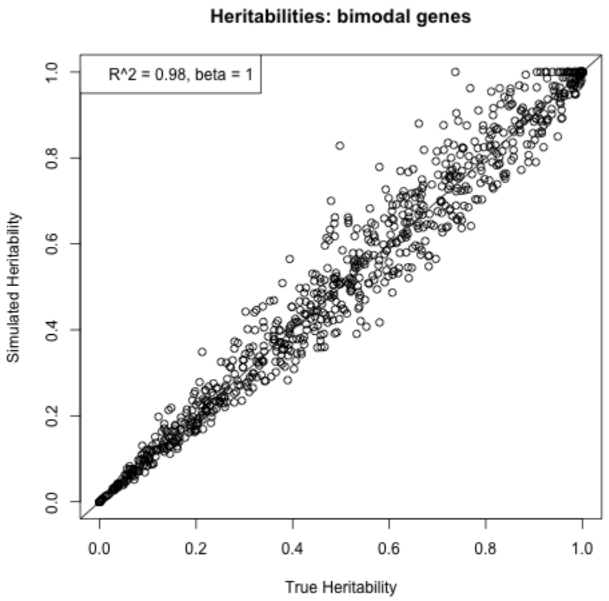

Figure S3: Simulated chip-based heritability versus true chip-based heritability for 1000 simulated bimodal genes (simulation 2).

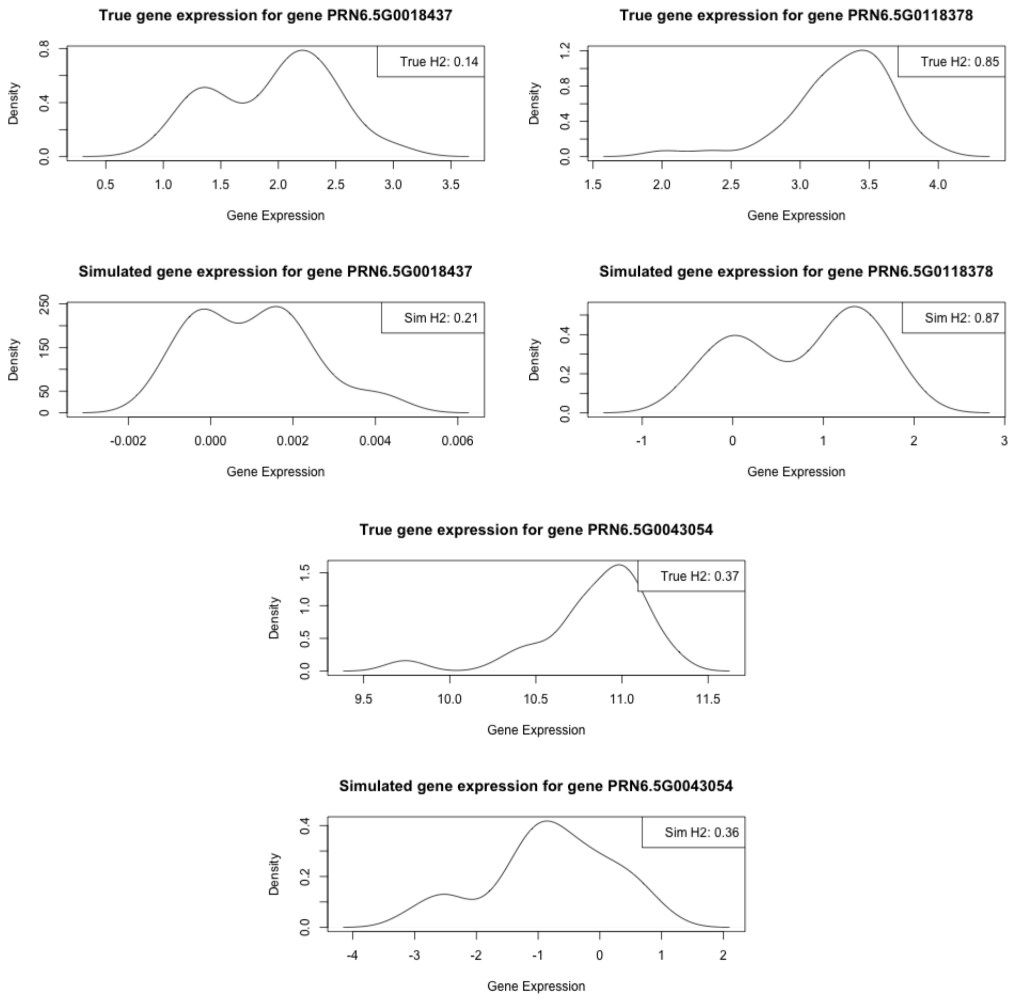

Figure S4: Distribution of simulated versus true gene expression for three randomly chosen bimodal genes (simulation 2).

Cluster Assignments	Unimodal	Bimodal	Trimodal
Count of Genes	760	222	18

Table S3: Count of genes per number of modes as estimated by 'mclust', for bimodal simulation (simulation 2).

S1.3 Multiple cis effect simulation

The setup of the multiple cis effect simulation is very similar to the setup of the single cis effect simulation, so many details will not be recapitulated here. The distinction is that the cis-SNPs are simulated from a different point-normal distribution, which is as follows:

$$\beta_j \sim_{iid} \begin{cases} N(0, \frac{h^2_{cis}}{p \times M_{cis}}), \text{ with probability p} \\ 0, \text{ with probability 1-p} \end{cases}$$

We fix p so that the expected number of cis-SNPs with nonzero effects is 2. That is, given M_{cis} which denotes the number of cis-SNPs in the gene, we define $p = 2/M_{cis}$. The rest of the quantities in the expression are the same as described in section **S1.2**.

Figure S5 shows that simulating effect sizes according to the above process does produce the desired heritabilities with no systemic bias. Figure S6 plots the distribution of the true and simulated phenotype

for three randomly selected genes. Table S4 displays the estimated modality of the simulated phenotypes for the 43-subject simulation. Again, the simulation induces a reasonable amount of bimodality, but a majority of the genes are estimated to be unimodal.

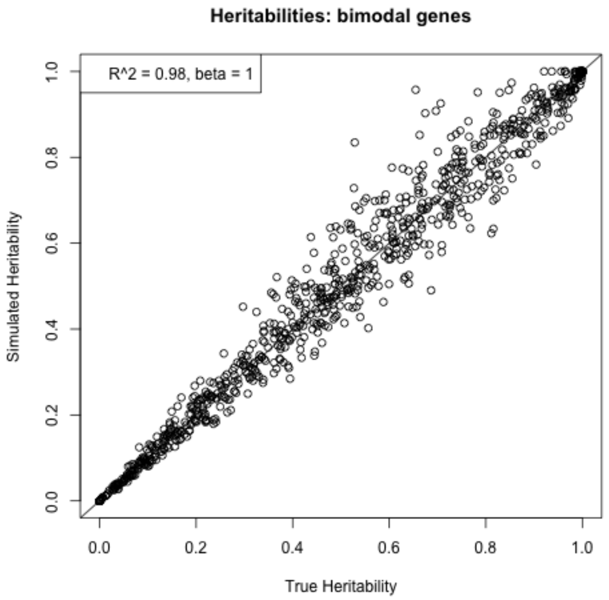

Figure S5: Simulated chip-based heritability versus true chip-based heritability for 1000 simulated bimodal genes (simulation 3).

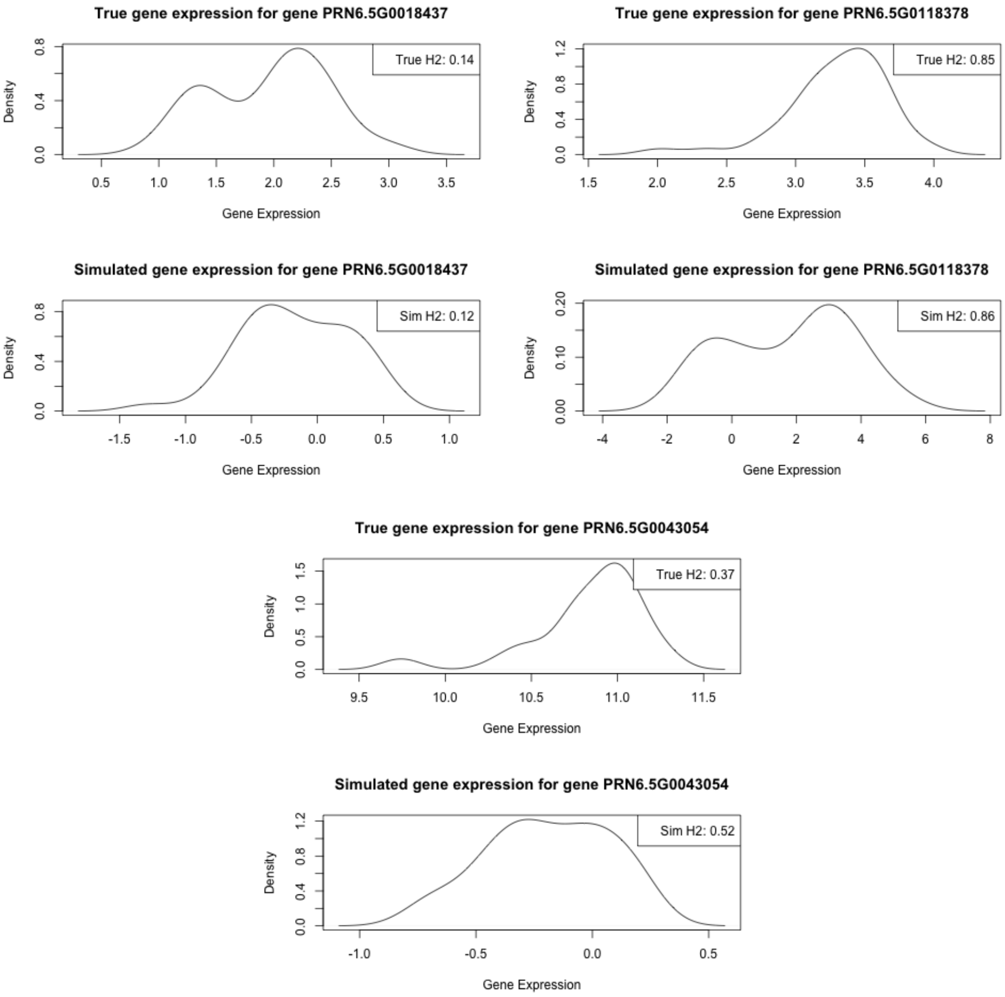

Figure S6: Distribution of simulated versus true gene expression for three randomly chosen bimodal genes (simulation 3).

Cluster Assignments	Unimodal	Bimodal	Trimodal
Count of Genes	703	290	7

Table S4: Count of genes per number of modes as estimated by 'mclust', for bimodal simulation (simulation 3).

S2 Supplementary Figures

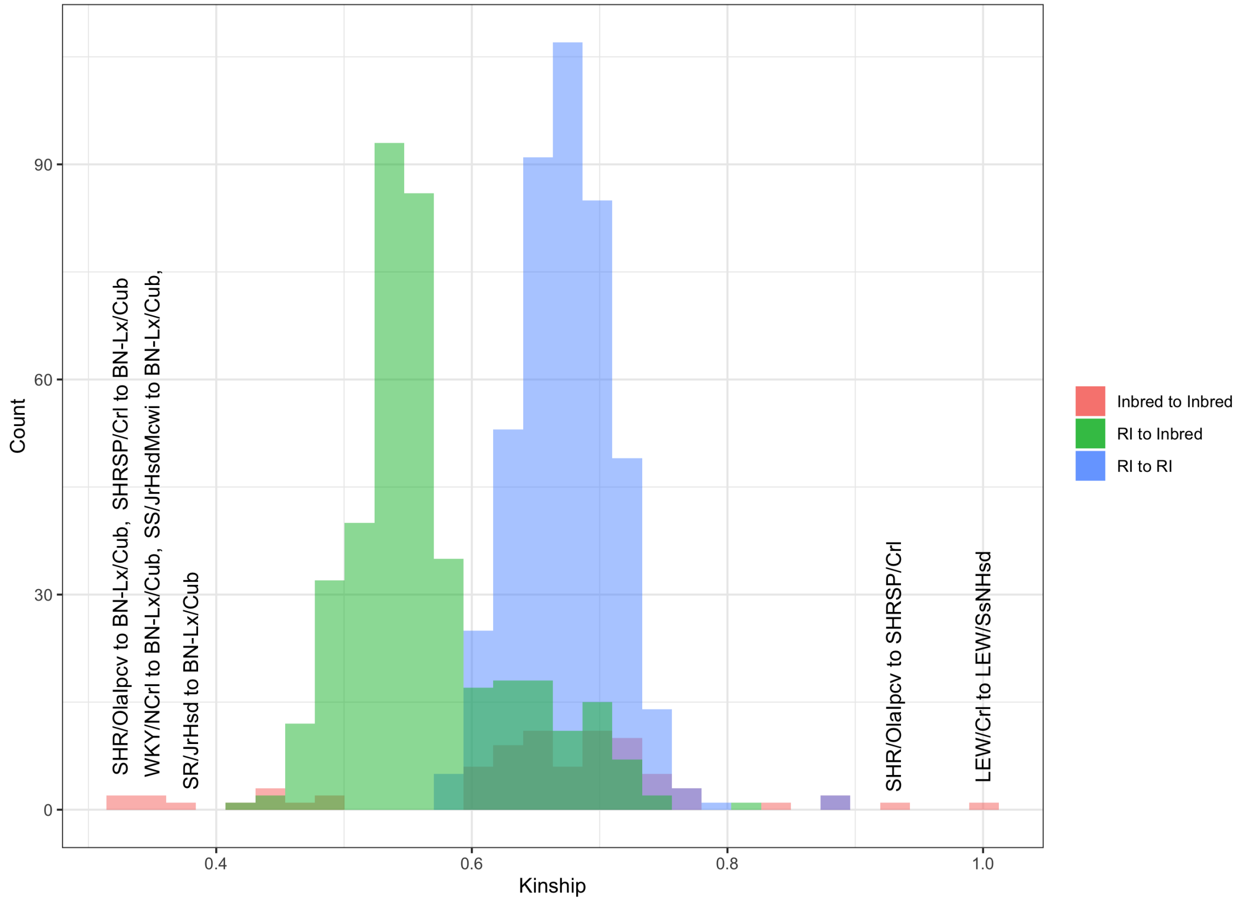

Supplementary Figure 1: Kinship values for each pairwise relationship between strains. Red denotes inbred to inbred kinship values, blue denotes RI to inbred kinship values and green denotes RI to RI kinship values. Extreme values are labeled with their corresponding pairs.

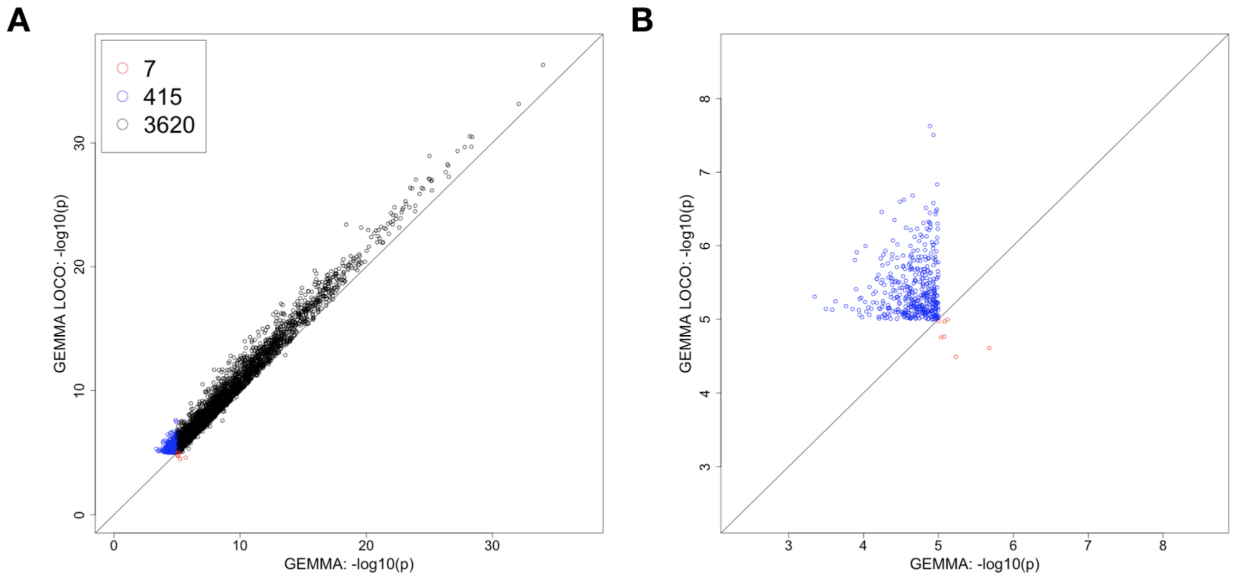

Supplementary Figure 2: Comparison of the negative base 10 logarithm of the p value of the most significant cis-SNP for the standard GEMMA (x axis) and GEMMA LOCO (y axis) models. Each point on the plot represents a gene with at least one cis-SNP identified as significant by at least one of the two methods. Panel A) shows associations that are significant in only GEMMA LOCO (blue), only GEMMA standard (red), and both (black). Panel B) shows only associations that are significant in exactly one analysis.

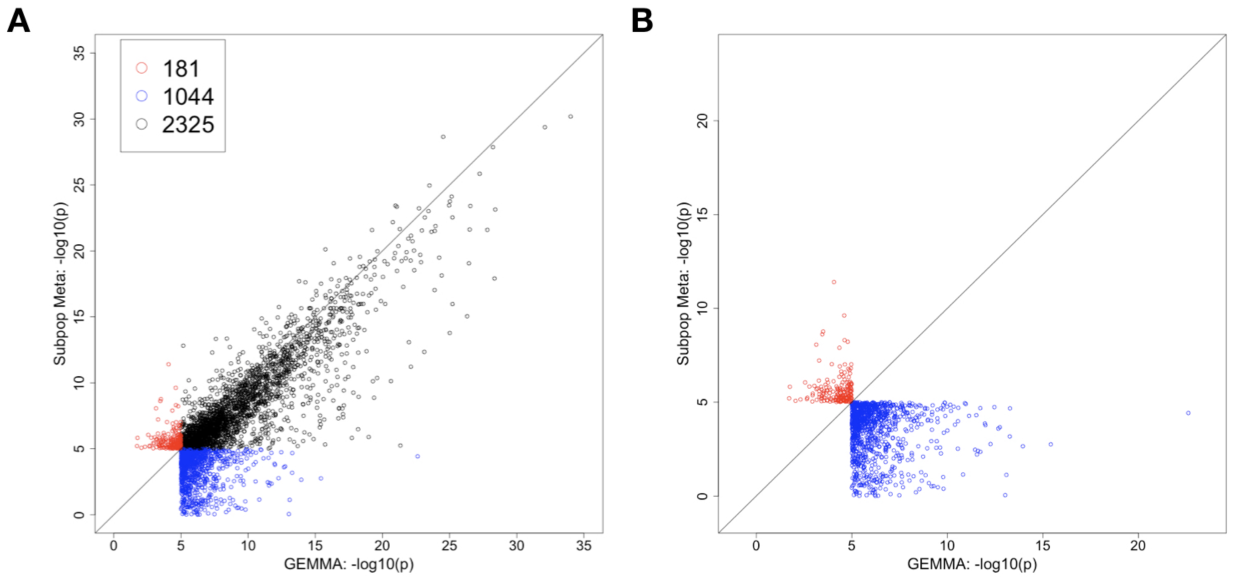

Supplementary Figure 3: Comparison of the negative base 10 logarithm of the p value of the most significant cis-SNP for the standard GEMMA (x axis) and subpopulation meta (y axis) models. Each point on the plot represents a gene with at least one cis-SNP identified as significant by at least one of the two methods. Panel A) shows associations that are significant in only GEMMA (blue), only in subpopulation meta (red), and both (black). Panel B) shows only associations that are significant in exactly one analysis.

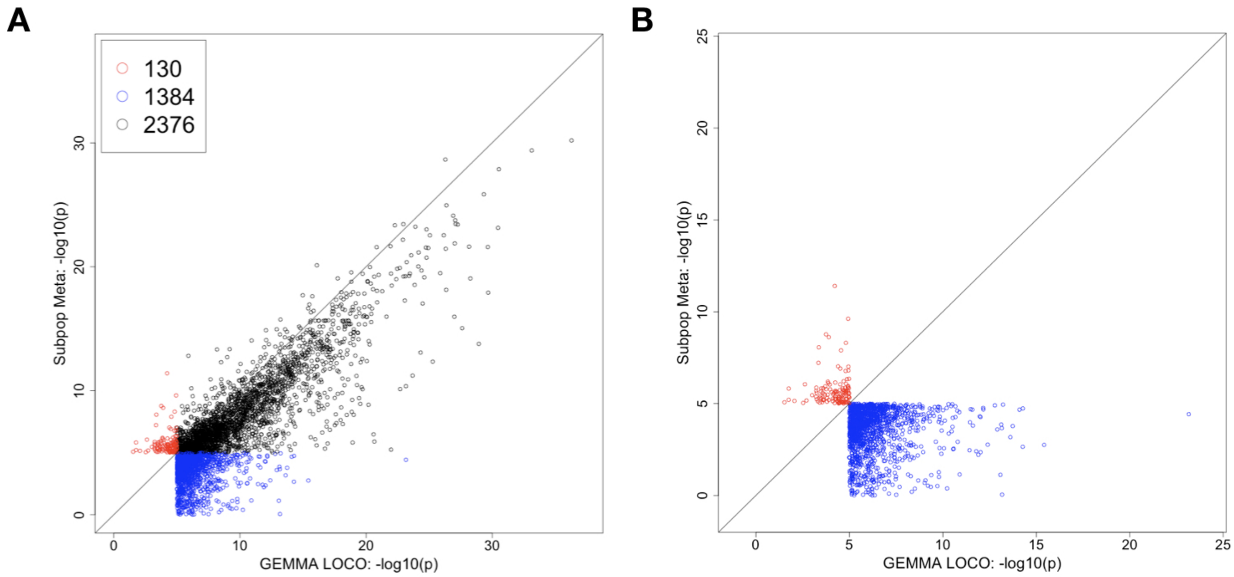

Supplementary Figure 4: Comparison of the negative base 10 logarithm of the p value of the most significant cis-SNP for the GEMMA LOCO (x axis) and subpopulation meta (y axis) models. Each point on the plot represents a gene with at least one cis-SNP identified as significant by at least one of the two methods. Panel A) shows associations that are significant in only GEMMA LOCO (blue), only in subpopulation meta (red), and both (black). Panel B) shows only associations that are significant in exactly one analysis.

S3 Supplementary Tables

	Minimum	1st Quartile	Median	Mean	3rd Quartile	Maximum
GEMMA	0.34	1.00	1.13	1.12	1.25	2.45
GEMMA LOCO	0.34	1.07	1.22	1.19	1.33	2.27
Subpopulation Meta	0.45	0.98	1.09	1.10	1.20	2.66
Linear Regression	1.94	3.01	3.78	4.48	5.34	15.08

Supplementary Table 1: The distribution of the genomic inflation factor across 99,736 eQTL transcripts for each of the four methods.

Method	Type I Error
GEMMA	0.008
GEMMA LOCO	0.010
Subpopulation Meta	0.015

Supplementary Table 2: Type I error for the three methods applied to the unimodal simulation for the 43-strain setting.

Subpopulation	Type I Error
FXLE/LEXF	0.014
HXB/BXH	0.025
Classic Inbred	0.037
All 92	0.041

Supplementary Table 3: Type I error for GEMMA LOCO analyses of the four subpopulations in unimodal simulation.

Method	Significant transcripts (cis correction)	Significant transcripts ($p < 10^{-5}$)	% significant
Standard GEMMA	11,917	2,925	18.5%
GEMMA LOCO	12,412	3,760	19.2%
Subpopulation Meta	10,886	2,506	16.9%
Linear Model	20,953	5,555	32.5%

Supplementary Table 4: The number of genes with at least one significant cis-SNP for each of the four methods, for only those genes with at least one cis-SNP for those SNPs which pass quality control in the subpopulation meta-analysis.

S4 Supplementary Details

S4.1 List of strains in the 43-strain subset

HXB1/Ipcv; 2307094
HXB2/Ipcv; 2307096
HXB3/Ipcv; 2307079
HXB4/Ipcv; 2307077
HXB5/Ipcv; 2307099
HXB7/Ipcv; 2307086
HXB10/Ipcv; 2307083
HXB13/Ipcv; 2307091
HXB15/Ipcv; 2307095
HXB17/Ipcv; 2307085
HXB18/Ipcv; 2307082
HXB20/Ipcv; 2307080
HXB21/Ipcv; 2307097
HXB22/Ipcv; 2307088
HXB23/Ipcv; 2307093
HXB24/Ipcv; 2307084
HXB25/Ipcv; 2307098
HXB27/Ipcv; 2307078
HXB29/Ipcv; 2307089
HXB31/Ipcv; 2307081
BXH2/Cub; 2307121
BXH3/Cub; 2307134
BXH5/Cub; 2307115
BXH6/Cub; 2307136
BXH8/Cub; 2307126
BXH9/Cub; 2307129
BXH10/Cub; 2307124
BXH11/Cub; 2307127
BXH/12-1/Cub; 2307139
BXH13/Cub; 2307123
ACI-SegHsd; 737892
Cop-CrCrl; 1358153
DA-OlaHsd; 734475
F344-Stm; 1302686
LE-Stm; 629485
LEW-Crl; 737932
LEW-SsNHsd; 737922
SHRSP-A3NCrl; 2311051
SR-JrHsd; 1582184
SS-JrHsdMcwi; 61499
WKY-NCrl; 1358112
BN-Lx/Cub; 61117

SHR-Olalpcv; 631848
